# Factors contributing to uncertainty in paediatric abdominal ultrasound reports in the paediatric emergency department

**DOI:** 10.1186/s12873-023-00892-w

**Published:** 2023-10-10

**Authors:** Soyun Hwang, Hyun Jung Chung, Joong Wan Park, Eui Jun Lee, Ha Ni Lee, Jin Hee Kim, Jie Hee Jue, Young Hun Choi, Jae Yun Jung

**Affiliations:** 1 Department of Pediatrics, Yonsei School of Medicine, Severance Children’s Hospital, Seoul, Republic of Korea; 2 Department of Pediatrics, Konkuk University Hospital, Seoul, Republic of Korea; 3 Department of Emergency Medicine, Seoul National University Hospital, 101, Daehak-Ro, Jongno-gu, Seoul, 03080 Republic of Korea; 4 Department of Radiology, Seoul National University Hospital, Seoul, Republic of Korea

**Keywords:** Ultrasonography, Emergency department, Paediatrics

## Abstract

**Background:**

Abdominal pain, which is a common cause of children presenting to the paediatric emergency department (PED), is often evaluated by ultrasonography (US). However, uncertainty in US reports may necessitate additional imaging.

**Objective:**

In this study, we evaluated factors contributing to uncertainty in paediatric abdominal US reports in the PED.

**Materials and methods:**

This retrospective cohort study included children younger than 18 years of age who underwent abdominal US in the PED of the study hospital between January 2017 and December 2019. After exclusion, the researchers manually reviewed and classified all US reports as ‘certain’ or ‘uncertain’. Univariate and multivariate logistic regression analyses were performed to identify the factors contributing to uncertain reports.

**Results:**

In total, 1006 patients were included in the final analysis., 796 patients were tagged as having certain reports, and 210 as having uncertain reports. Children with uncertain reports had a significantly higher rate of undergoing an additional computed tomography (CT) scan (31.0% vs. 2.5%, p < 0.001) and a longer PED median length of stay (321.0 (Interquartile range (IQR); 211.3-441.5) minutes vs. 284.5 (IQR; 191.8-439.5) minutes, p = 0.042). After logistic regression, US performed by a radiology resident (odds ratio, 5.01; 95% confidence interval, 3.63–7.15) was the most significant factor contributing to uncertainty in paediatric abdominal US reports followed by obesity and age.

**Conclusion:**

Several factors contribute to uncertainty in paediatric abdominal US reports. Uncertain radiological reports increase the likelihood of additional CT scans. Measures to improve the clarity of radiological reports must be considered to improve the quality of care for children visiting the PED.

**Supplementary Information:**

The online version contains supplementary material available at 10.1186/s12873-023-00892-w.

## Introduction

Abdominal pain is one of the most common causes of paediatric emergency department (PED) visits, accounting for 3–8% of total PED visits [1]. However, the differential diagnosis of abdominal pain in children is challenging, and in many cases, radiological studies, such as x-rays, ultrasound (US), and computed tomography (CT), are required.

Radiologic studies are among the most effective tools for diagnosing diseases and making effective treatment strategies for patients, and the number of radiologic studies performed annually in the ED is increasing [2–4]. However, because radiation exposure may increase the risk of malignancy in children, there is a movement (As Low As Reasonably Achievable; ALARA) to minimize radiation exposure in children as much as possible [5]. Therefore, to improve paediatric health, many efforts have been made to reduce radiation exposure in the diagnosis of intra-abdominal pathologies, such as diagnosing acute appendicitis by ultrasound. Despite the increased use of ultrasound and technological advances, the interpretation of ultrasound results may differ, sometimes with uncertain results.

In a previous study, the recommendation for additional radiological studies increased the use of follow-up CT imaging in children suspected of having appendicitis [6]. In addition, agreement between radiologists and referring physicians regarding diagnostic certainty has been poor, with reports containing ambiguous words, such as ‘unlikely’, ‘suspicious for’, and ‘possibly’, which can lead to over-testing and/or over-imaging [7–9]. Therefore, it is crucial to identify the factors that lead to uncertain radiologic reports in order to improve the quality of care provided to children in the PED. However, current studies on additional CT imaging after ultrasound have primarily focused on patient-related factors, such as the patient’s history, laboratory values, and physical examination findings, rather than US reports, [10].

In this study, we evaluated the factors contributing to uncertainty in paediatric abdominal US reports that led to additional CT scans in the PED. In addition, we measured the amount of radiation exposure caused by the additional CT scans.

## Methods

### Ethics statement

This study was conducted in accordance with the principles of the Declaration of Helsinki and approved by the Institutional Review Board (IRB) of Seoul National University Hospital. The need for informed consent was waived by the IRB of Seoul National University Hospital owing to the retrospective nature of the study and minimal risk to the patients.

### Study design and setting

This retrospective cohort study was conducted at Seoul National University Hospital, a tertiary-level urban teaching hospital where approximately 18,000 children visit the PED annually. All patients visiting the PED were initially evaluated and treated by a resident emergency physician and an attending paediatric emergency specialist. Whenever a child required an imaging workup, the attending paediatric emergency specialist determined the primary imaging modality and requested an examination from the radiologist. The US examination was performed by a paediatric radiology specialist during working hours (weekdays, 9 AM to 6 PM) and by a radiology resident at other times, such as nights or holidays. In our hospital, we do not strictly observe a fasting period for imaging study in emergency department, and our radiologists prioritize conducting ultrasounds as promptly as possible.

### Methods and measurements

Children under 18 years of age who underwent an abdominal US examination in the PED of the study hospital between January 2017 and December 2019 were eligible. During the COVID-19 pandemic, there were many changes regarding the treatment process in the PED; therefore, the study was limited to the pre-pandemic period. Children were excluded if they had a previous abdominal surgery, were treated for a chronic intra-abdominal disease (such as malignancy or hepatobiliary disease), or underwent a CT scan prior to the US examination. Patients who had incomplete height or weight information at the time of their PED visit were excluded from the analysis. During study period, we identified 21 revisits within three days from initial visit. Among these, 14 visits were attributed to recurring abdominal pain following the initial discharge of intussusception with a successful reduction, while the remaining seven were linked to worsening abdominal pain following an initial visit with a normal ultrasound. However, all of these revisit cases occurred more than 24 h after the initial visit, leading us to consider them as individual visits. Also, the possibility of ovarian pathology contributing to lower abdominal pain in a female child was not excluded.

We extracted a list of eligible patients and variables from the study hospital’s clinical data warehouse. Patient information, such as age, sex, weight, height, underlying diseases, date and time of PED visit and discharge, PED length of stay (LOS), chief complaint, and PED result (admission, discharge, or transfer), was collected. The chief complaints were categorized into three distinct groups for analysis: ‘abdominal symptoms,’ which encompassed complaints such as abdominal pain, vomiting, diarrhea, or bloody stool; ‘fever,’ designated for cases evaluated to determine the source of fever without specific mention of abdominal symptoms; and ‘others,’ reserved for complaints like irritability, excessive crying, or general weakness, without specific mention of definite abdominal pain or fever. The body habitus of each child was calculated and categorised using a Z-score of growth indicators according to the World Health Organization’s child growth standards [11, 12] (body mass index [BMI] for age or weight for age if height was not measured). Radiology-related variables, such as the time of the US examination, the performing radiologist (either a resident or a specialist), the US and subsequent CT scan report (if available), and the volume CT dose index (CTDI_vol_) and dose-length product (DLP) of each CT scan, were also collected. In addition, we recorded whether urgent surgical or radiological intervention (e.g., appendectomy or air reduction of intussusception) was required within 24 h for children who underwent additional CT scans due to an uncertain radiologic report, and retrieved final surgical diagnosis if possible.

Three paediatric emergency specialists manually reviewed each initial US examination report. Uncertain reports were defined as those that did not fully diagnose or exclude a disease. Such reports included phrases like ‘limited study, target organ (e.g., the appendix) was not fully visualised’; ‘CT scan may be helpful under clinical suspicion’; ‘uncertain findings, follow-up imaging is recommended’; or ‘target disease (e.g., appendicitis) cannot be excluded’ (Table 1). The reason for uncertainty in the radiologic reports, such as ‘obesity’, ‘abundant bowel gases’, ‘patient was too irritable’, or ‘poor sonic window’, was tagged. If no specific reason for uncertainty was stated in the radiology report, the reason was tagged as ‘unknown’. Furthermore, if the radiologist explicitly indicates that an adequate imaging study could not be conducted due to insufficient fasting time or incomplete bladder filling, it was categorized as ‘nonadherence to protocol.‘

Mean DLP values were compared with commonly used, published, age- and region-specific conversion coefficients (Supplementary Table 1) to estimate the effective dose of radiation, according to the 1990 recommendations of the International Commission on Radiological Protection (ICRP) [13, 14].

### Outcome measures

The primary outcome of this study was the identification of factors that contribute to uncertainty in radiologic reports. The secondary outcome was the incidence of additional CT scans after the initial US examination in each group.

### Statistical analysis

Continuous variables were presented as medians with interquartile ranges, and categorical variables were presented as numbers and percentages. Normality testing was performed using the Shapiro–Wilk test to compare variables between patient groups. As the data were skewed from normality, continuous variables were compared using the Mann–Whitney U test, and categorical variables were compared using the Chi-square test. To identify factors that led to an uncertain report, univariate and multivariate logistic regressions with stepwise selection of the variable were performed after multicollinearity identification using variance inflation factors (VIF). Statistical significance was set at P < 0.05. All statistical analyses were performed using R, version 4.0.2 (R Foundation for Statistical Computing, Vienna, Austria).

## Results

**Fig. 1:**
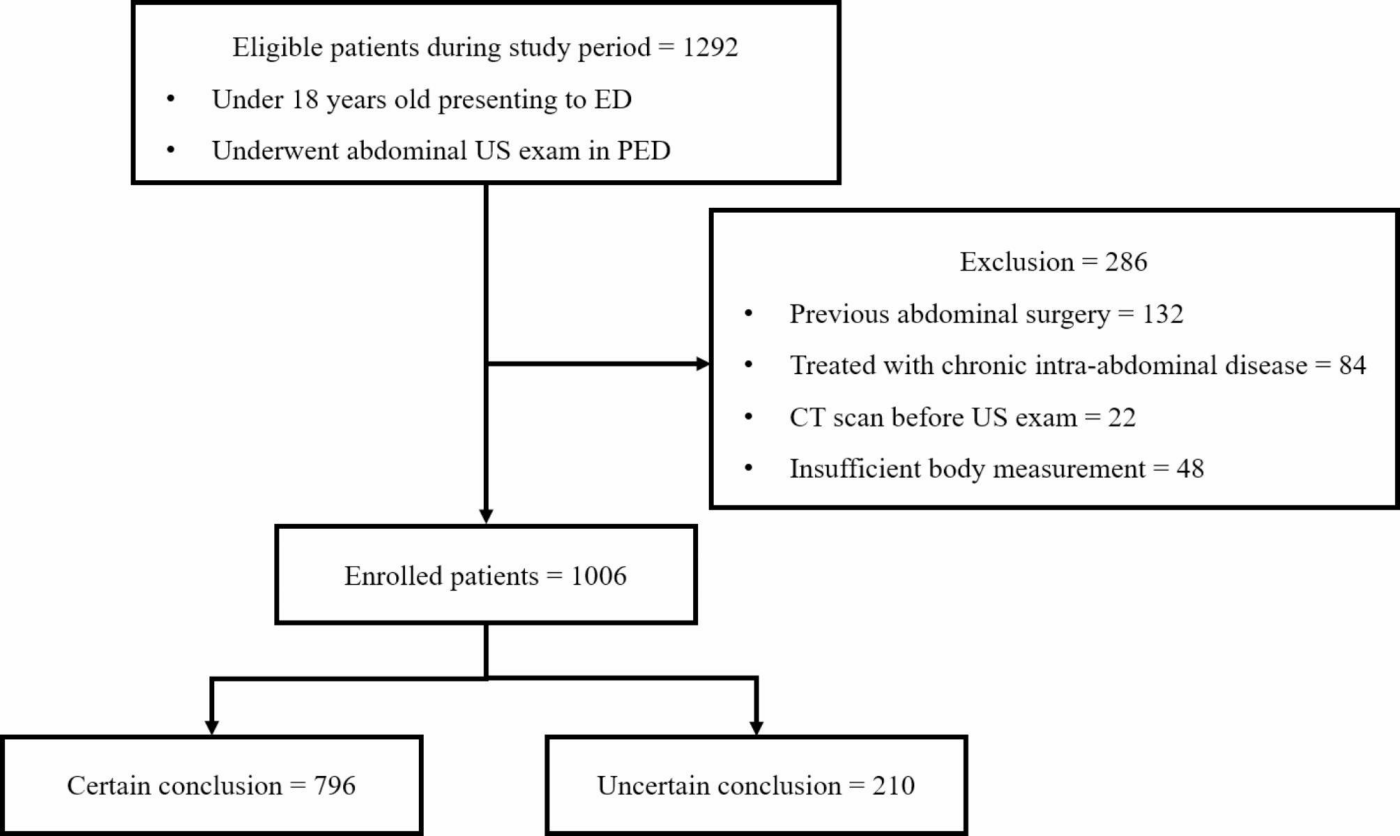
Flowchart of study participants

Of the 1292 eligible patients, 132 were excluded due to previous abdominal surgery, 84 due to chronic intra-abdominal disease, 22 due to a CT scan prior to their US examination, and 48 due to insufficient body measurement. After exclusion, 1006 patients were included in the final analysis (Fig. 1). After the radiology report review, 796 (79.1%) patients were tagged with certain reports, and 210 (20.9%) patients were tagged with uncertain reports. The patient characteristics and outcome variables are shown in Table 1. The sex and chief complaints of both groups were not significantly different, but children with uncertain reports were older and weighed more. Children with uncertain reports had a higher rate of undergoing US performed by a radiology resident (65.7% vs. 30.7%, *p* < 0.001) and additional CT scans (31.0% vs. 2.5%, *p* < 0.001). Although there was no significant difference in LOS, the PED results were significantly different, with a higher rate of admissions or transfers in children with certain reports and a higher rate of discharges and three against medical advice exclusively observed with uncertain reports. Urgent interventions were more frequent in children with certain reports than in those with uncertain reports (25.7% vs. 18.3%, *p* = 0.002). Among those who required urgent interventions, the majority of children (93.1%) received discharge diagnoses which is concordant with their initial ultrasound radiology reports. Only two patients exhibited discordance between the initial ultrasound radiology report and surgical confirmation, and notably, these instances occurred exclusively with uncertain reports.

**Table 1:** Demographics of study participants^a^

	Overall	Certain reports	Uncertain reports	*p*
**N**	1006	796	210	
**Boys**	567 (56.4)	460 (57.8)	107 (51.0)	0.089
**Additional CT**	85 (8.4)	20 (2.5)	65 (31.0)	< 0.001
**Reason for additional CT** **Clinician decision** **Uncertain report** **Next step**	6 (7.0) 65 (75.6) 15 (17.4)	6 (28.6) 0 (0.0) 15 (71.4)	0 (0.0) 65 (100.0) 0 (0.0)	< 0.001
**Ultrasound by residents**	382 (38.0)	244 (30.7)	138 (65.7)	< 0.001
**Chief complaint** **Abdominal symptoms** **Fever** **Others**	750 (74.6) 113 (11.2) 143 (14.2)	601 (75.5) 88 (11.1) 107 (13.4)	149 (71.0) 25 (11.9) 36 (17.1)	0.338
**ED disposition** **Transfer out** **Admission** **AMA discharge** **Discharged**	17 (1.7) 344 (34.2) 3 (0.3) 642 (63.8)	15 (1.9) 285 (35.8) 0 (0.0) 496 (62.3)	2 (1.0) 59 (28.1) 3 (1.4) 146 (69.5)	0.001
**Urgent intervention**	232 (23.1)	201 (25.3)	31 (14.8)	0.002
**Surgical confirmation** **Concordant** **Discordant** **Transfer out**	216 (93.1) 2 (0.9) 14 (6.0)	189 (94.0) 0 (0.0) 12 (6.0)	27 (87.1) 2 (6.5) 2 (6.5)	0.001
**Age (months)**	52.2 [17.3, 92.6]	47.5 [15.9, 87.6]	65.8 [21.5, 108.0]	0.001
**ED LOS (minutes)**	292.0 [197.0, 440.5]	284.5 [191.8, 439.5]	321.0 [211.3, 441.5]	0.042
**Body weight (kg)**	16.05 [10.70, 25.85]	15.93 [10.50, 24.30]	18.00 [12.00, 33.53]	0.001
**Body habitus** **Overweight/obesity**	200 (19.9)	146 (18.3)	54 (25.7)	0.022

^**a**^Values are presented as the number (%) or median [IQR]

AMA = Against Medical Advice ; CT = computed tomography; ED = emergency department; LOS = length of stay

Among the 210 uncertain reports, the most common reason for uncertainty was ‘abundant bowel gas’ (71 cases, 33.81%), followed by ‘unknown’ (60 cases, 28.57%) and ‘irritable child’ (47 cases, 22.38%). However, nine cases were tagged as uncertain reports because of equivocal findings, such as the borderline diameter of the appendix, rather than a suboptimal study (Table 2).

**Table 2:** Reasons for uncertain reports

Category	N	%
Abundant bowel gas	71	33.81%
Unknown	60	28.57%
Irritable child	47	22.38%
Poor sonic window	19	9.05%
Equivocal finding	9	4.29%
Nonadherence to protocol	4	1.90%
Total	210	100.00%

**Fig. 2:**
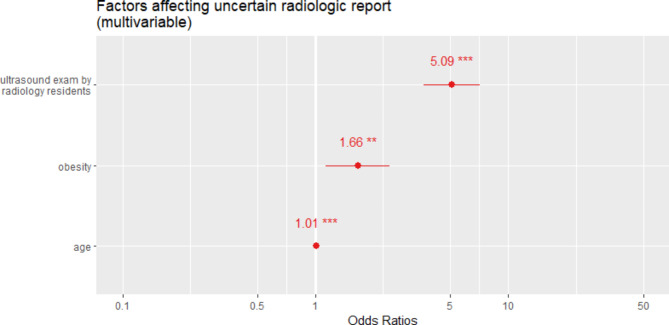
Forest plot of factors contributing to uncertain radiologic reports

After univariate regression (Table 3) and tests for multicollinearity using VIF, multivariate logistic regression with stepwise regression was performed (Table 4; Fig. 2). US performed by a radiology resident (odds ratio [OR], 5.01; 95% confidence interval [CI], 3.63–7.12), body weight (OR, 1.66; 95% CI, 1.132–2.43), and age (OR, 1.01; 95% CI, 1.005–1.012) were significantly associated with uncertain reports.

In children who underwent additional CT scans because of an uncertain report, the median CTDI_vol_ was 2.38 mGy (95% CI, 1.42–3.54), and that of DLP was 95 mGycm (95% CI, 51–145). The median effective dose calculated according to age was 1.73 mSv (95% CI, 1.13–2.36).

**Table 3:** Univariable logistic regression

	Odds ratio	2.50%	97.50%	*p*
Ultrasound by a resident	4.336	3.141	5.985	< 0.001
Body habitus – overweight/obesity	1.541	1.078	2.204	0.018
Chief complaint – others	1.357	0.893	2.061	0.152
Chief complaint - fever	1.146	0.71	1.85	0.577
Age (months)	1.005	1.002	1.008	< 0.001
Boys	0.759	0.559	1.029	0.076

**Table 4:** Multivariable logistic regression

	Odds ratio	2.50%	97.50%	*p*
Ultrasound by a resident	5.093	3.630	7.147	< 0.001
Body habitus – overweight/obesity	1.658	1.132	2.430	< 0.001
Age (months)	1.008	1.005	1.012	< 0.001

## Discussion

In this study, the factors contributing to uncertainty in abdominal US reports in children visiting the PED were US examinations by a radiology resident, body habitus, and age. In addition, uncertain radiological reports significantly increased the rate of children undergoing additional CT scans.

The most important factor leading to uncertain radiologic reports was US examinations by a radiology resident. Previous studies have shown discrepancies between radiology residents’ preliminary radiology reports and faculty members’ formal reports [15]. Body habitus was also an important factor because the rate of uncertain reports increased in obese group. Obesity or a high BMI is a well-known factor that reduces the diagnostic ability of abdominal US in both adults and children [16, 17]. There are previous studies that reports decreased sensitivity of ultrasound diagnosing appendicitis in obese/overweight children [18–20], and Sulowski et al. [21] emphasized the significance of reexamination and reimaging in cases of obese children suspected of having appendicitis and undergoing screening abdominal ultrasound.

A higher number of additional CT scans were performed in cases with uncertain radiological reports, whereas a higher rate of children underwent urgent intervention if they had certain reports. Among the 210 patients with uncertain reports, 65 (31.0%) underwent CT due to uncertain radiologic reports. Only 18 of the 65 children needed urgent intervention within 24 h, suggesting that a large number of children received unnecessary radiation exposure due to uncertain radiologic reports. In contrast, among the 796 children who had certain radiological reports, only 20 (2.5%) underwent an additional CT scan due to medical necessity (such as evaluation for metastasis of a newly diagnosed malignancy). Therefore, although 13 of the 20 children did not require urgent intervention, it cannot be assumed that radiation exposure was unnecessary. In this study, additional CT scans due to uncertain radiological reports resulted in a median additional effective radiation dose of 1.73 mSv per child. Larger radiation doses and a younger age at exposure increase the lifetime risk of cancer [22–24]. The effective radiation dose of 1.73 mSv is less than the annual exposure to background radiation (approximately 3.0 mSv) [25] but up to 500 times higher than that of simple chest x-rays [26].

In a previous study, 74% of children with suspected appendicitis who underwent abdominal US had non-definitive conclusions, 60% had disclaimers, and 25% of those patients underwent a subsequent CT scan. Among the non-definitive conclusions, when the CT scan was performed because of the disclaimer, positive appendicitis was observed in 29% of cases [6]. In this study, the proportion of uncertain reports was smaller (20.87%) than that in a previous study, whereas 31.0% of patients had additional CT scans, which is similar to that of the previous study.

In our study, the most common reason for uncertainty in radiologic reports was ‘abundant bowel gas’ (33.81%), followed by ‘not confident’ (28.57%) and ‘irritable child’ (22.38%). While it is plausible that our hospital’s practice of not adhering to fasting times before conducting ultrasounds may play a role in generating uncertain radiologic reports due to bowel gas, it’s important to note that gas-generated reverberation artefacts are a well-known cause of US artifacts [27, 28], and adequate training for techniques, such as graded compression, can help improve study quality [28, 29]. Nevertheless, the exact prevalence of these artifacts remains poorly documented. Additionally, hydrosonography can be considered for specific indications [30].

Radiological reports are the most important means of communication between radiologists and clinicians. However, radiologists with less experience than specialists may have limitations when performing and reading abdominal US images. This may explain why many patients did not receive a confident examination by a radiology resident. Residents may be unable to find the target organ as well as the specialist, and even if they do find the target organ, they may report uncertainty with the intention of protecting themselves from medicolegal problems due to a lack of confidence in their interpretation. Furthermore, radiology reports can be interpreted differently by different treating physicians [7, 8]. Phrases like ‘probably’ and ‘unlikely’, which are commonly used in radiology reports, do not represent exact probabilities and may confuse physicians.

However, it is important for children to receive consistent, quality care, regardless of day or night. It would be ideal to have certified paediatric radiologists working 24 h a day; unfortunately, not every hospital has a full-time, certified paediatric radiologist, and in many institutions, radiology residents interpret radiologic studies after working hours. Several methods can be considered to reduce uncertain radiological reports and improve the quality of care during PED off-hours.

Encouraging education and proper training of paediatric radiologists are important for improving the quality of US imaging performed by radiology residents. Previous studies have shown that hospitals with more paediatric patients or a paediatric-focused ED had significantly lower CT scan rates for abdominal pain [31, 32] due to better accessibility and more experience, emphasizing the importance of proper training. In addition, standardization of radiology reporting, such as a structured report template, can be considered [33, 34] for better communication between radiologists and treating physicians. Furthermore, it is important that physicians provide radiologists with accurate clinical information. Adopting a low-dose CT protocol can also help prevent excessive radiation exposure [35].

Our study had some limitations. First, because this study was a retrospective study conducted in a single hospital, we could not follow up with individual children. If a child was discharged from the PED and never returned, we could not determine whether the child had a true surgical diagnosis that required intervention without a repeat visit to our hospital. Given that a substantial number of children in our study population were discharged, we recommend that future research consider a prospective approach, possibly involving outpatient clinic follow-ups or phone-call monitoring, to address this limitation. Additionally, potential bias from the treating PED physicians or caregivers was not considered. In our hospital, although emergency medicine residents and paediatric emergency medicine specialists work in pairs and are responsible for making important decisions, the decision to order an additional CT scan might have been affected by the previous experience or personal preference of each specialist, or even the patient density of the PED at the time. In addition, if caregivers were especially anxious or if it was difficult for the patient to visit the hospital several times, they might have wanted to obtain a definitive result from a single visit. These problems were difficult to overcome owing to the retrospective design of this study, and further research into these factors is needed in the future. Additionally, we did not include children with a history of previous abdominal surgery or those with chronic intra-abdominal pathology, despite this potentially representing a significant portion of the pediatric patients seen in the PED. However, detailed demographic information and the reasons for uncertain conclusions regarding these excluded children can be found in Supplementary Tables 2 and 3.

US examination by a radiology resident was the most important factor contributing to uncertainty in abdominal US reports in the paediatric population. Uncertain radiological reports increase the likelihood of additional CT scans. Measures to improve the clarity of radiological reports must be considered to improve the quality of care for children visiting the PED.

### Electronic supplementary material

Below is the link to the electronic supplementary material.

Supplementary Material 1

## Data Availability

The datasets used and analysed in the current study are available from the corresponding author upon reasonable request.
