# Supplementary material for: Factors contributing to uncertainty in paediatric abdominal ultrasound reports in the paediatric emergency department
Source: BMC Emerg Med. 2023 Oct 10;23:120. doi: 10.1186/s12873-023-00892-w (PMC10566031; doi:10.1186/s12873-023-00892-w)
Supplement: Supplementary file 1 — Supplementary Material 1 [file 12873_2023_892_MOESM1_ESM.docx]

**Supplement table 1. The value of conversion factor that are used in this study**

| Region of body | 0 year old | 1 year old | 5 year old | 10 year old | Adult |
| --- | --- | --- | --- | --- | --- |
| Head and Neck | 0.013 | 0.0085 | 0.0057 | 0.0042 | 0.0031 |
| Head | 0.011 | 0.0067 | 0.0040 | 0.0032 | 0.0021 |
| Neck | 0.017 | 0.012 | 0.011 | 0.0079 | 0.0059 |
| Chest | 0.039 | 0.026 | 0.018 | 0.013 | 0.014 |
| Abdomen and Pelvis | 0.049 | 0.030 | 0.020 | 0.015 | 0.015 |
| Trunk | 0.044 | 0.028 | 0.019 | 0.014 | 0.015 |

**Supplement table 2. Demographics of excluded children due to abdominal surgery or previously known intra-abdominal pathology.**

|  | **Overall** | **Certain reports** | **Uncertain reports** | ***p*** |
| --- | --- | --- | --- | --- |
| **N** | 216 | 153 | 63 |  |
| **Boys** | 104 (48.1) | 79 (51.6) | 25 (39.7) | 0.148 |
| **Additional CT** | 19 (8.8) | 8 (5.2) | 11 (17.5) | 0.009 |
| **Reason for additional CT**  **Clinician decision**  **Uncertain report**  **Next step** | 4 (21.1)  11 (57.9)  4 (21.1) | 4 (50.0)  0 (0.0)  4 (50.0) | 0 (0.0)  11 (100.0)  0 (0.0) | 0.011 |
| **Ultrasound by residents** | 45 (20.8) | 26 (17.0) | 19 (30.2) | 0.048 |
| **Prev. abdominal surgery** | 132 (61.1) | 92 (60.1) | 40 (63.5) | 0.759 |
| **Age (months)** | 83.3 [31.8, 143.7] | 79.1 [30.4, 141.6] | 99.8 [37.7, 148.2] | 0.568 |
| **ED LOS (minutes)** | 391.0 [284.3, 649.3] | 399.0 [279.0, 720.0] | 382.0 [303.0, 538.0] | 0.617 |

**Supplement table 3. Reason for uncertain conclusions in excluded children due to abdominal surgery or previously known intra-abdominal pathology.**

| Category | N | % |
| --- | --- | --- |
| bowel gas | 33 | 52.38% |
| poor sonic window | 15 | 23.81% |
| unknown | 11 | 17.46% |
| irritable child | 4 | 6.35% |
| Total | 63 | 100.00% |
